# A dual-barrier sustained-release hydrogel based on Cu-exchanged Y zeolite and sodium alginate for infected wound healing

**DOI:** 10.1039/d6ra08040e

**Published:** 2026-11-12

**Authors:** Ying Rao, Xiaofang Yu, Kunlan Xu, Lifeng Zheng, Shuhua Pu, Na Wen, Pengzhao Wang, Wei Lin

**Affiliations:** a Department of Otolaryngology Head and Neck Surgery, The First Affiliated Hospital of Fujian Medical University Fuzhou 350005 P. R. China guaguatong@126.com; b College of Chemical Engineering, Fuzhou University Fuzhou 350108 P. R. China wpz@fzu.edu.cn; c Department of Anesthesiology, Fuzhou University Affiliated Provincial Hospital Fuzhou 350116 P. R. China; d Department of Pathology, Fuzhou University Affiliated Provincial Hospital Fuzhou 350116 P. R. China; e Department of Orthopedics, The First Affiliated Hospital of Fujian Medical University Fuzhou 350005 P. R. China; f College of Materials Science and Engineering, Fuzhou University Fuzhou 350116 P. R. China; g Department of Otolaryngology Head and Neck Surgery, National Regional Medical Center, Binhai Campus of the First Affiliated Hospital of Fujian Medical University Fuzhou 350212 China; h Fujian Institute of Otorhinolaryngology, The First Affiliated Hospital of Fujian Medical University Fuzhou 350005 China; i Fujian Clinical Research Center for Difficult Otorhinolaryngologic Diseases, The First Affiliated Hospital of Fujian Medical University Fuzhou 350005 China

## Abstract

Bacterial wound infection and the growing crisis of antibiotic resistance urgently demand non-antibiotic wound dressings that integrate long-term antibacterial activity and high biosafety. Herein, we designed a dual-barrier sustained-release antibacterial hydrogel (CuY@SA) by embedding Cu^2+^-exchanged Y zeolite (CuY) into a calcium-crosslinked sodium alginate (SA) matrix. The ordered microporous zeolite acts as the first-stage reservoir to enable intrinsic Cu^2+^ sustained release *via* ion exchange, while the alginate network serves as a second physical diffusion barrier to suppress burst release and prolong delivery. The resulting CuY@SA hydrogel eliminated the initial burst and achieved nearly-linear Cu^2+^ release over 48 h, with an equilibrium swelling ratio of approximately 28 g g^−1^ in pH 7.4 PBS. *In vitro*, CuY@SA exhibited over 99.9% bactericidal efficacy against *Escherichia coli* and *Staphylococcus aureus*, while maintaining >98% cytocompatibility with NIH-3T3 fibroblasts at hydrogel concentrations up to 10 mg mL^−1^. In a mouse full-thickness infected wound model, CuY@SA significantly accelerated wound closure to over 98% by day 14, promoted collagen deposition and re-epithelialization, reduced local inflammation, and caused no obvious organ toxicity or cuproptosis. Compared with the control hydrogel (Cu@SA) containing directly added free Cu^2+^, CuY@SA showed remarkably lower cytotoxicity and superior *in vivo* antibacterial activity, confirming the critical role of zeolite in precise controlled release. This work demonstrates that combining an inorganic zeolite carrier with a biopolymer hydrogel provides a simple, scalable, and effective strategy for developing safe and long-acting antibacterial wound dressings. More broadly, it highlights the untapped potential of zeolites as versatile and tunable controlled-release platforms for advanced biomedical applications.

## Abstract 2

Bacterial wound infection and the growing crisis of antibiotic resistance urgently demand non-antibiotic wound dressings that integrate long-term antibacterial activity and high biosafety.

## Graphical abstract

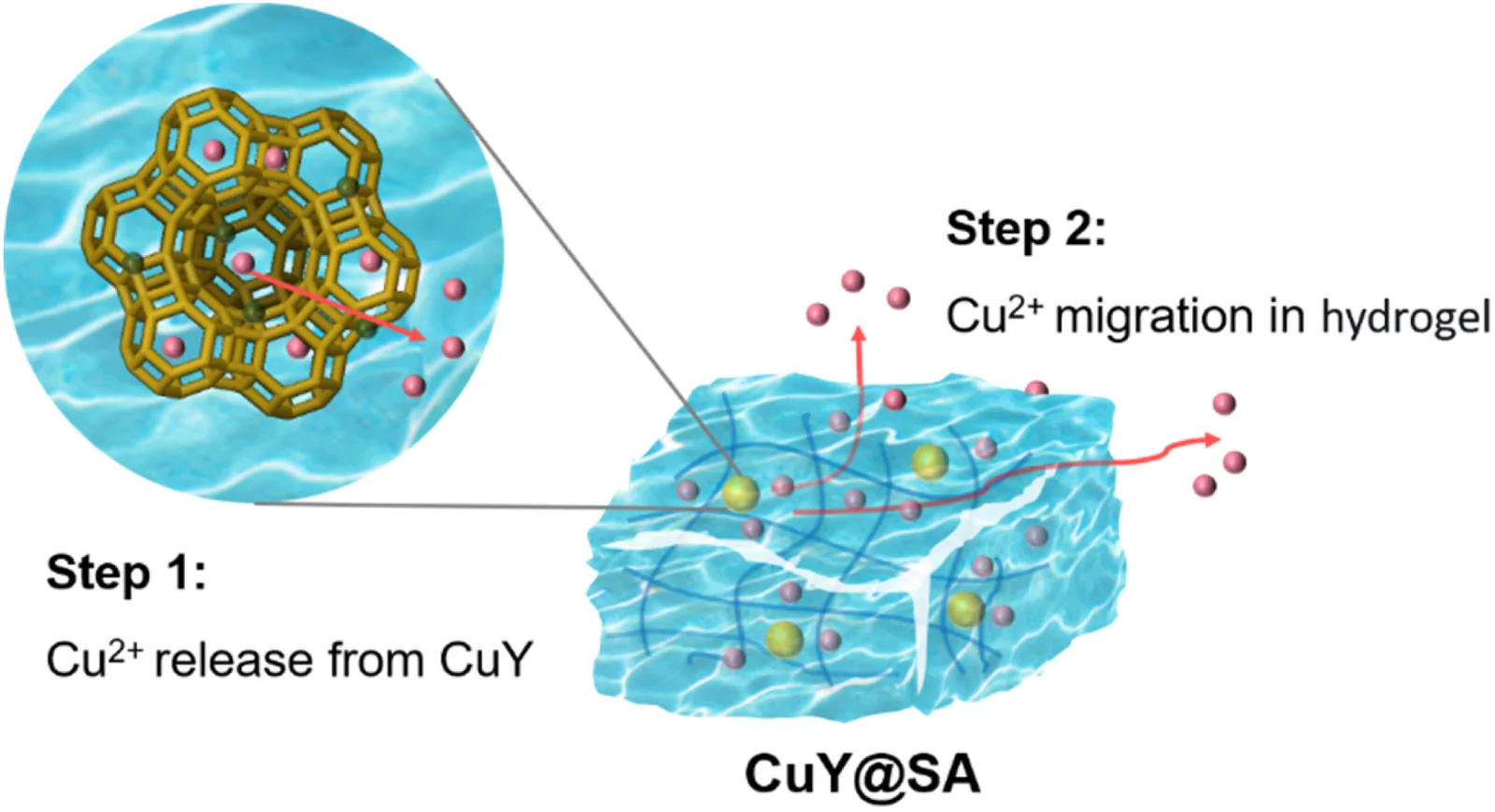

## 1. Introduction

Bacterial infection remains one of the most serious challenges in wound care, often leading to delayed healing, chronic inflammation, and even life-threatening complications.^1–3^ Traditional antibacterial therapies rely mainly on antibiotics, but their long-term and excessive use has accelerated the rise of drug-resistant bacteria, while systemic administration frequently results in insufficient local drug levels and systemic side effects.^4–6^ These limitations have driven extensive research toward non-antibiotic antibacterial materials that can provide safe, long-acting, and locally effective treatment.^7–10^ Among the many alternatives to antibiotics, metal ions such as Ag^+^,^11^ Zn^2+^,^12^ and Cu^2+^ (ref. 13) have drawn particular interest due to their broad-spectrum antibacterial action and low risk of inducing resistance. Cu^2+^ is often preferred because it is inexpensive, highly effective, and, as an essential trace element in the human body, potentially safer than non-physiological metal ions when used within an appropriate concentration range.^14,15^

However, the clinical translation of Cu^2+^-based antibacterial materials faces a serious problem: free Cu^2+^ ions released in an uncontrolled burst not only shorten the duration of antibacterial action,^16^ but also risk local cytotoxicity.^14,17^ Excessive Cu^2+^ accumulation induces oxidative stress and mitochondrial damage, which severely compromises biosafety *in vivo*.^18,19^ Both antibacterial activity and cytotoxicity of Cu^2+^ exhibit obvious concentration-dependent behavior. Burst release produces an abrupt local Cu^2+^ spike that exceeds the tolerance threshold of normal cells, triggering non-specific oxidative injury and aggravating local inflammatory complications. In contrast, controlled ion release maintains Cu^2+^ concentration within a therapeutic window, sufficient for bactericidal effects while remaining tolerable to host cells.^20,21^ Therefore, a controlled-release carrier is urgently required to achieve sustained Cu^2+^ delivery and avoid burst-induced toxicity.^22,23^ An effective carrier is therefore needed to store Cu^2+^ and release it slowly over time.

Zeolites are crystalline aluminosilicates with a uniform microporous structure, large specific surface area, and excellent ion-exchange capacity.^24–26^ These properties make them well suited as carriers for antibacterial metal ions.^27^ Cu^2+^ can be loaded into the zeolite framework by replacing the native charge-balancing cations,^23,28^ and the narrow pore channels slow down ion diffusion, providing an intrinsic first-stage sustained release. Beyond ion exchange, the regular pore architecture of zeolites also allows encapsulation of larger therapeutic molecules, giving them broad potential as drug delivery platforms.^29–32^ Recent studies have shown that zeolitic imidazolate frameworks (ZIFs), a related class of microporous materials, can be combined with bacterial cellulose hydrogels to create antibacterial composites with enhanced antimicrobial properties.^33^ Despite these advantages, zeolites have received less attention in biomedical research compared to organic or polymer-based carriers,^24,34^ and their potential as ion-release controllers in wound dressings remains largely unexplored.^35^

Hydrogels are three-dimensional crosslinked polymer networks that can absorb and retain large amounts of water while maintaining their structure.^36–38^ Sodium alginate (SA), a natural polysaccharide extracted from brown algae,^39,40^ is widely exploited in wound dressings for cutaneous wound repair owing to its excellent biocompatibility, mild gelation conditions, and capacity to generate a moist microenvironment conducive to tissue healing.^41,42^ Importantly, the dense polymer network of a hydrogel can act as a physical barrier that slows the outward diffusion of loaded agents, offering a second level of control over the release rate.^40^ Combining an inorganic zeolite carrier with a biopolymer hydrogel therefore creates a dual-barrier system in which the zeolite provides the first release barrier and the hydrogel network provides the second.^43^ Nevertheless, pristine sodium alginate still faces shortcomings including poor mechanical performance and uncontrollable degradation, which can be mitigated *via* chemical modification, polymer blending or inorganic filler hybridization for drug delivery and wound-healing applications.^44–46^ Various characterization approaches have been adopted to evaluate the mechanical suitability of hydrogel dressings; for instance, Shore A hardness serves as a practical indentation-based metric for compact cast hydrogel films.^47^

Inspired by the above considerations, we hypothesize that integrating Cu-exchanged Y zeolite with calcium-crosslinked sodium alginate can construct a tandem dual-barrier system to mitigate the undesirable burst release of Cu^2+^. This architecture is expected to sustain Cu^2+^ delivery within a safe therapeutic window, balancing potent antibacterial activity and cytocompatibility for infected wound treatment. Accordingly, the present work aims to fabricate the CuY@SA composite hydrogel and systematically characterize its structural and physicochemical properties. We further assess its ion-release behavior, *in vitro* antibacterial performance, cytocompatibility, as well as *in vivo* healing efficacy and biosafety in a mouse full-thickness infected wound model.

## 2. Materials and methods

### 2.1 Materials

NaY zeolite (SiO_2_/Al_2_O_3_ = 5.4) was obtained from Nankai University Catalyst Co., Ltd. Cu(NO_3_)_2_·3H_2_O, CaCl_2_, NaCl, sodium alginate (SA, BR., molecular weight: ∼100 kDa, viscosity: 200 ± 20 mPa s for a 1% (w/v) solution at 25 °C, product no. S817372), beef extract (BR), agar (BR), and tryptone (BR) were purchased from Shanghai Macklin Biochemical Technology Co., Ltd. Phosphate-buffered saline (PBS) was purchased from Shanghai Life iLab Biochemical Technology Co., Ltd. All reagents were used without further purification. *Staphylococcus aureus* (*S. aureus*) and *Escherichia coli* (*E. coli*) strains were supplied by Guangdong Huankai Microbial Technology Co., Ltd. Cell Counting Kit-8 (CCK-8) was purchased from Invigentech Inc.

### 2.2 Preparation of CuY and CuY@SA

Synthesis of CuY. CuY was prepared by ion exchange. Typically, 1.00 g of NaY zeolite was dispersed in 10 mL of 0.1 mol L^−1^ Cu(NO_3_)_2_ solution and stirred at 80 °C for 2 h. The solid was collected by centrifugation, washed with deionized water until the supernatant reached neutral pH, and dried at 100 °C for 12 h. The ion-exchange step was repeated twice to achieve sufficient copper loading. The resulting powder was then calcined at 550 °C for 4 h to obtain the final CuY sample.

Preparation of CuY@SA. CuY@SA hydrogel was prepared by embedding CuY particles into an alginate network crosslinked by Ca^2+^. Briefly, 0.10 g of CuY powder was dispersed in 14 g of deionized water under ultrasonication for 15 min. The suspension was heated to 60 °C, and 0.42 g of SA was added under stirring. After the SA had fully dissolved, 2 mL of CaCl_2_ solution (0.135 mol L^−1^) was added dropwise to trigger ionic crosslinking of the alginate chains, forming a three-dimensional network that physically trapped the CuY particles. The hydrogel was cooled to room temperature and rinsed thoroughly with deionized water to remove unbound ions, yielding the CuY@SA hydrogel. Brief rinsing with deionized water induces slight swelling driven by electrostatic repulsion of alginate carboxylate groups, but the primary ionic crosslinking network remains intact. Since swelling tests were conducted with lyophilized xerogels, water uptake during rinsing exerts no influence on swelling results.

Two control hydrogels were prepared under the same conditions: (i) SA hydrogel, without CuY, serving as a blank matrix; and (ii) Cu@SA hydrogel, in which CuY was replaced by an equivalent molar amount of Cu(NO_3_)_2_. In Cu@SA, Cu^2+^ ions were directly coordinated to the alginate chains rather than being pre-loaded in a zeolite carrier, allowing evaluation of the role of the zeolite in controlling Cu^2+^ release. Photographs of as-prepared CuY powder, CuY@SA and Cu@SA hydrogels are provided in Fig. S1 of the SI for visual reference.

### 2.3 Characterizations of NaY and CuY

X-ray diffraction (XRD) was performed on a D8 Advance diffractometer (Bruker, Germany) to analyze the crystal structure of samples. The measurement was carried out with Cu Kα radiation (*λ* = 0.15406 nm) at 40 kV and 40 mA, scanning from 5° to 40° at 5° min^−1^. Fourier-transform infrared (FTIR) spectra were recorded using a Nicolet IS50 spectrometer (Thermo Scientific, USA). FTIR measurements were performed in attenuated total reflectance (ATR) mode. Spectra were collected in the range of 4000–400 cm^−1^ with 32 scans and a resolution of 4 cm^−1^ to identify the structural groups of the samples. Scanning electron microscopy (SEM) images were acquired on a Verios G4 ultra-high-resolution field-emission scanning electron microscope (Thermo Scientific, USA) to observe the micro-morphology of samples. All samples were sputter-coated with gold before observation. X-ray fluorescence (XRF) analysis was conducted on a ZSX Primus spectrometer (Rigaku, Japan) to determine the bulk elemental composition of zeolites. The pressed pellet samples were tested, and the elemental contents were quantified according to the characteristic X-ray intensities of each element. Nitrogen adsorption–desorption measurements were performed using an ASAP2460 analyzer (Micromeritics, USA) to characterize the pore structure of zeolite samples. H_2_ temperature-programmed reduction (H_2_-TPR) tests were carried out on a BSD-C200 chemisorption analyzer to evaluate the distribution of copper species. Briefly, 50 mg of sieved sample (20–40 mesh) was pre-treated at 800 °C under Ar flow for 1 h and then cooled to 50 °C. The atmosphere was switched to 5% H_2_/Ar, and the sample was heated to 1000 °C at a rate of 10 °C min^−1^ and held for 30 min. The TCD signal was recorded as a function of temperature. Zeta potential and dynamic light scattering (DLS) particle-size measurements were performed on a NanoPlus 3 analyzer. Samples were homogeneously dispersed in ultrapure water prior to testing. Inductively coupled plasma optical emission spectrometry (ICP-OES, Avio 200, PerkinElmer, USA) was used to quantify the Cu^2+^ concentration in liquid supernatants, with the detection wavelength set at 327 nm.

### 2.4 Swelling behavior

Lyophilized hydrogels, xerogels obtained by pre-freezing wet hydrogels in liquid nitrogen followed by vacuum freeze-drying, were weighed and immersed in PBS at 37 °C. The swelling ratio was calculated as:Swelling ratio = (*M*_s_ − *M*_0_)/*M*_0_where *M*_0_ and *M*_s_ represent the initial weight and swollen weight, respectively. Swelling measurements were performed at pH 7.4, which serves as a physiological reference condition for bodily fluids in acute-wound-related environments.

### 2.5 Rheological measurements

Rheological properties were measured on an Anton Paar MCR301 rheometer using a parallel plate geometry (diameter 30 mm, gap 1 mm). Strain sweep (1 Hz, 0.001–100% strain) and frequency sweep (0.01% strain, 1–100 rad s^−1^) tests were performed to evaluate the mechanical stability and gel strength.

### 2.6 *In vitro* Cu^2+^ release

The samples were separately immersed in 100 mL of release medium (deionized water or pH 7.4 PBS buffer) and incubated in a thermostatic water bath at 37 °C. At predetermined time-points, 1 mL of supernatant was withdrawn for analysis, and an equal volume of pre-warmed fresh release medium was replenished to maintain constant system volume. For ICP-OES measurement, collected supernatants were pre-diluted with 2% nitric acid. Copper concentrations in the release media were quantified against external calibration standards, and each sample was analyzed in triplicate.

### 2.7 Antibacterial activity

The antibacterial activity was evaluated using the spread plate method. Samples were co-cultured with *S. aureus* and *E. coli* (1 × 10^6^ CFU per mL) for 12 h at 37 °C. The antibacterial rate was calculated as:
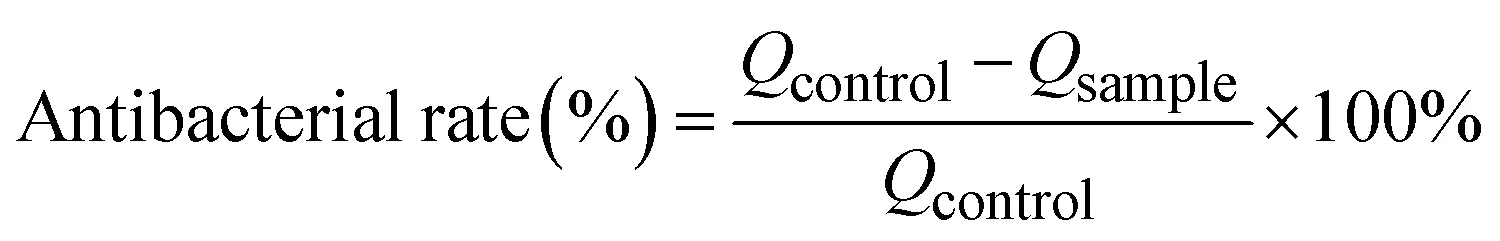
where *Q*_control_ and *Q*_sample_ represent the colony counts of the control and sample groups, respectively.

### 2.8 Cytocompatibility

NIH-3T3 fibroblasts were seeded into 96-well plates at a density of 6 × 10^3^ cells per well and cultured for 24 h. To obtain hydrogel extracts, the hydrogels were incubated in DMEM at 37 °C for 24 h. The extracts were then added to the cells. After co-culture for 24 h and 48 h at 37 °C in a 5% CO_2_ atmosphere, cell viability was measured using the CCK-8 assay and calculated as:
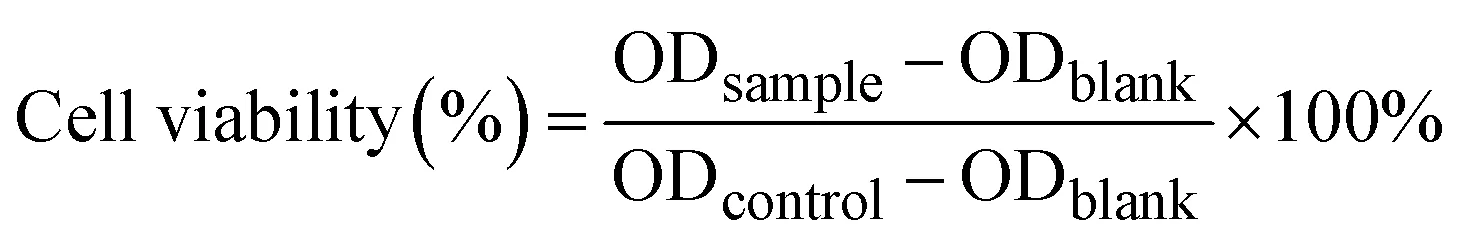
where OD_blank_, OD_control_ and OD_sample_ represent the absorbance of the blank, control and sample groups, respectively.

### 2.9 *In vivo* wound healing study

All animal experiments were approved by the Animal Ethics Committee of the First Affiliated Hospital of Fujian Medical University (IACUC FJMU 2025-Y-1298). All procedures were performed in accordance with the guidelines of the Institutional Animal Care and Use Committee and relevant national research standards. Mice were housed at 22–25 °C under a 12 h light–dark cycle with free access to food and drinking water. Full-thickness infected wound models were established in male ICR mice (6–8 weeks old, SPF grade). The mice were anesthetized with isoflurane, the dorsal hair was shaved, and the skin was disinfected. A circular full-thickness wound (8 mm in diameter) was created on the dorsum, and 10 µL of *S. aureus* suspension (1 × 10^6^ CFU per mL) was inoculated onto the wound. All wounds were covered with a transparent film dressing (3M Tegaderm). After 24 h, the infected mice were randomly divided into three groups (*n* = 6 per group): (i) PBS (control), (ii) Cu@SA hydrogel, and (iii) CuY@SA hydrogel. The hydrogels were applied to cover the wound and replaced every three days. Wound images were taken on days 0, 3, 6, 9, and 14, and the wound area was measured using ImageJ software. The wound healing rate was calculated as:
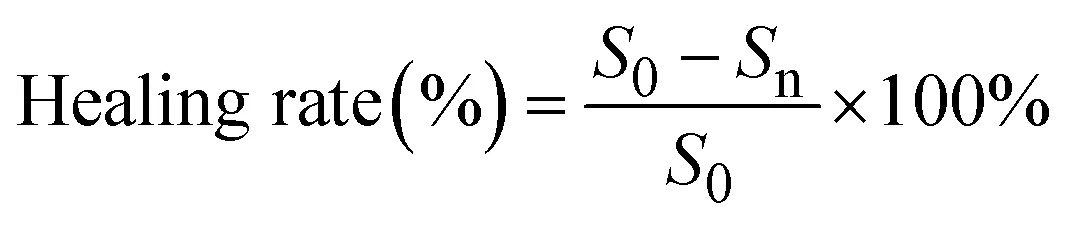
where *S*_0_ is the initial wound area and *S*_n_ is the wound area at each time point.

### 2.10 Statistical analysis

Data were expressed as mean ± standard deviation. Differences were analyzed by one-way ANOVA with Tukey's post-hoc test. All experiments were performed in triplicate. Significance levels: *P < 0.05, **P < 0.01, ***P < 0.001, ****P < 0.0001. Unless comparison pairs are labelled in figures, asterisks denote comparisons between the tested group and the control group.

## 3. Results and discussion

### 3.1 Characterization and Cu^2+^ release behavior of CuY

#### 3.1.1 Structure and morphology of CuY

The XRD pattern of CuY shows all the characteristic reflections of the FAU structure, matching those of the parent NaY zeolite (Fig. 1a). No peaks from crystalline CuO or other copper oxides were found, indicating that the copper species are well dispersed inside the zeolite framework rather than forming large particles on the external surface. The diffraction peaks of CuY are slightly weaker than those of NaY, which is likely due to partial dealumination of the framework during ion exchange and calcination.

**Fig. 1:**
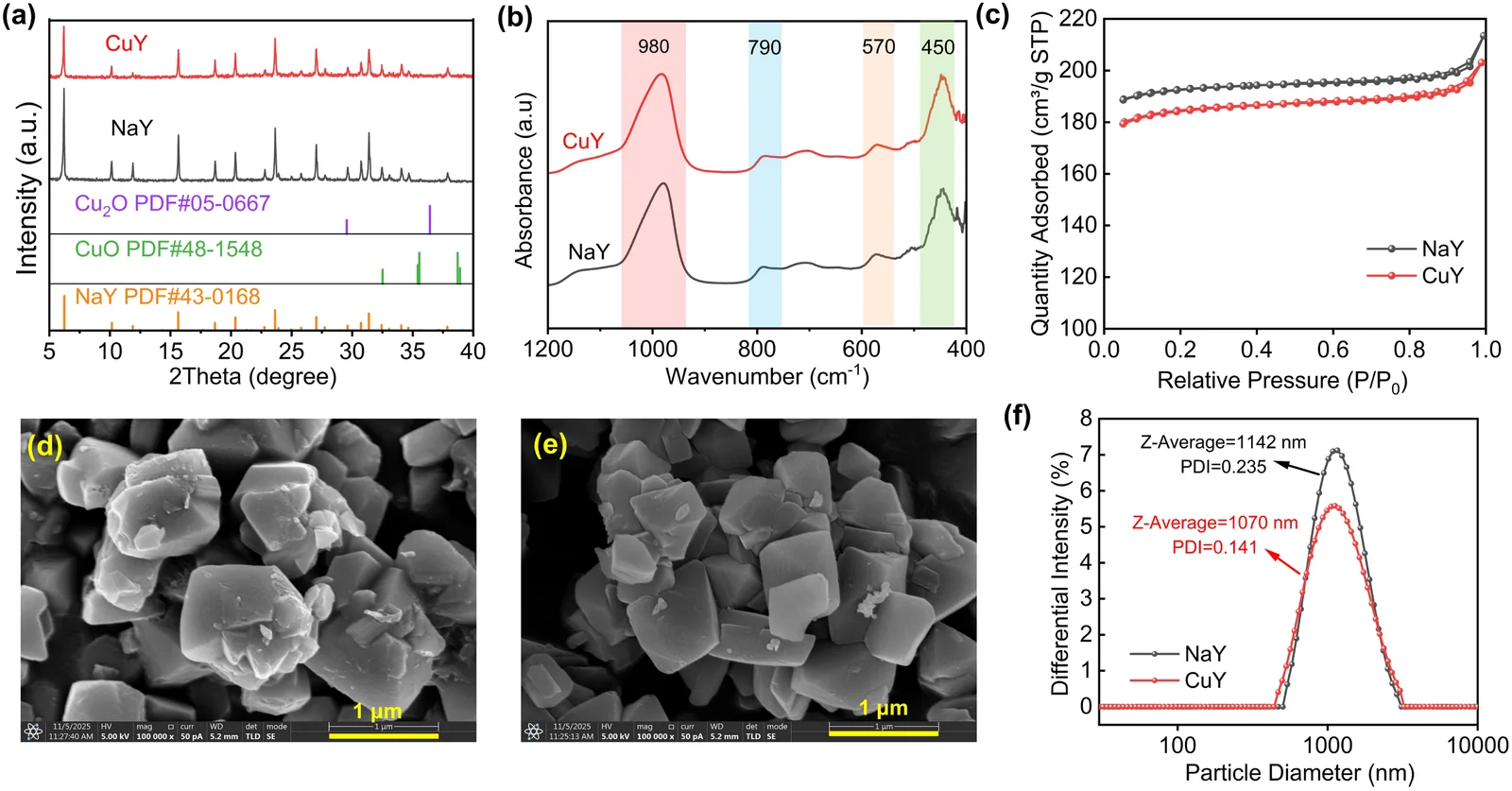
Structural and morphological characterization of NaY and CuY. (a) XRD patterns. (b) FT-IR spectra. (c) N_2_ adsorption–desorption isotherms. SEM images of (d) NaY and (e) CuY. (f) Particle size distributions.

The FTIR spectra of NaY and CuY (Fig. 1b) further support the preservation of the zeolite framework. All the typical bands of the FAU structure are present:^48^ the asymmetric stretching of Si–O–T (T = Si or Al) near 980 cm^−1^, the symmetric stretching near 790 cm^−1^, the double six-membered ring (D6R) vibration near 570 cm^−1^, and the T–O bending mode near 450 cm^−1^. No bands from copper oxide phases appeared, consistent with the XRD results.

The AlO-4 tetrahedra in the zeolite framework carry negative charges and must be balanced by cations. Based on charge balance, one Cu^2+^ can replace two Na^+^ during ion exchange. The elemental analysis by XRF (Table S1) gives a CuO content of 13.15% for CuY, which is close to the theoretical value of 12.57 wt%. This agreement confirms that most Cu^2+^ ions sit near the AlO_4_^−^ sites and are held there by strong electrostatic interactions. The Si/Al ratio of the parent NaY zeolite plays a decisive role in determining the maximum exchange capacity, as it directly dictates the density of framework negative charges available for cation exchange.^49^

The porosity and surface area of NaY and CuY were studied by N_2_ adsorption–desorption. Both samples showed a Type IV isotherm with an H3 hysteresis loop (Fig. 1c), typical of microporous materials with some mesopores. The Brunauer–Emmett–Teller (BET) surface area and pore volume remained nearly the same after ion exchange (Table S2), indicating that the loading of copper had little effect on the pore structure. This is important because the intact pore channels serve as pathways for Cu^2+^ release. The SEM images (Fig. 1d and e) show that CuY retains the same octahedral crystal shape as NaY, with a uniform particle size of approximately 1 µm. This observation agrees well with the DLS particle-size distribution measured using the NanoPlus 3 analyzer (Fig. 1f), and the corresponding PDI of CuY is 0.141. No obvious morphological change is found after ion exchange, demonstrating that the crystal framework of Y zeolite is well preserved. The zeta-potential result of CuY is shown in Fig. S2 of the SI.

#### 3.1.2 Cu^2+^ “Burst release” and antibacterial activity of CuY

The release behavior of Cu^2+^ from CuY was studied in deionized water and PBS at 37 °C under continuous stirring. As shown in Fig. 2a, the release curves display three distinct stages. In the first stage (0–3 h), the Cu^2+^ concentration rises rapidly, with about 45% of the total leachable copper released within the first 3 h. This is followed by a slower release phase (3–12 h), during which the Cu^2+^ concentration still increases but at a much lower rate. After 12 h, a plateau is reached, suggesting that the loosely bound and easily exchangeable Cu^2+^ ions have been largely depleted. In PBS, the overall Cu^2+^ concentration is lower than in water, which can be explained by the partial precipitation of Cu^2+^ with phosphate ions (PO_4_^3−^) in the buffer. Even so, the same three-stage pattern is clearly observed.

**Fig. 2:**
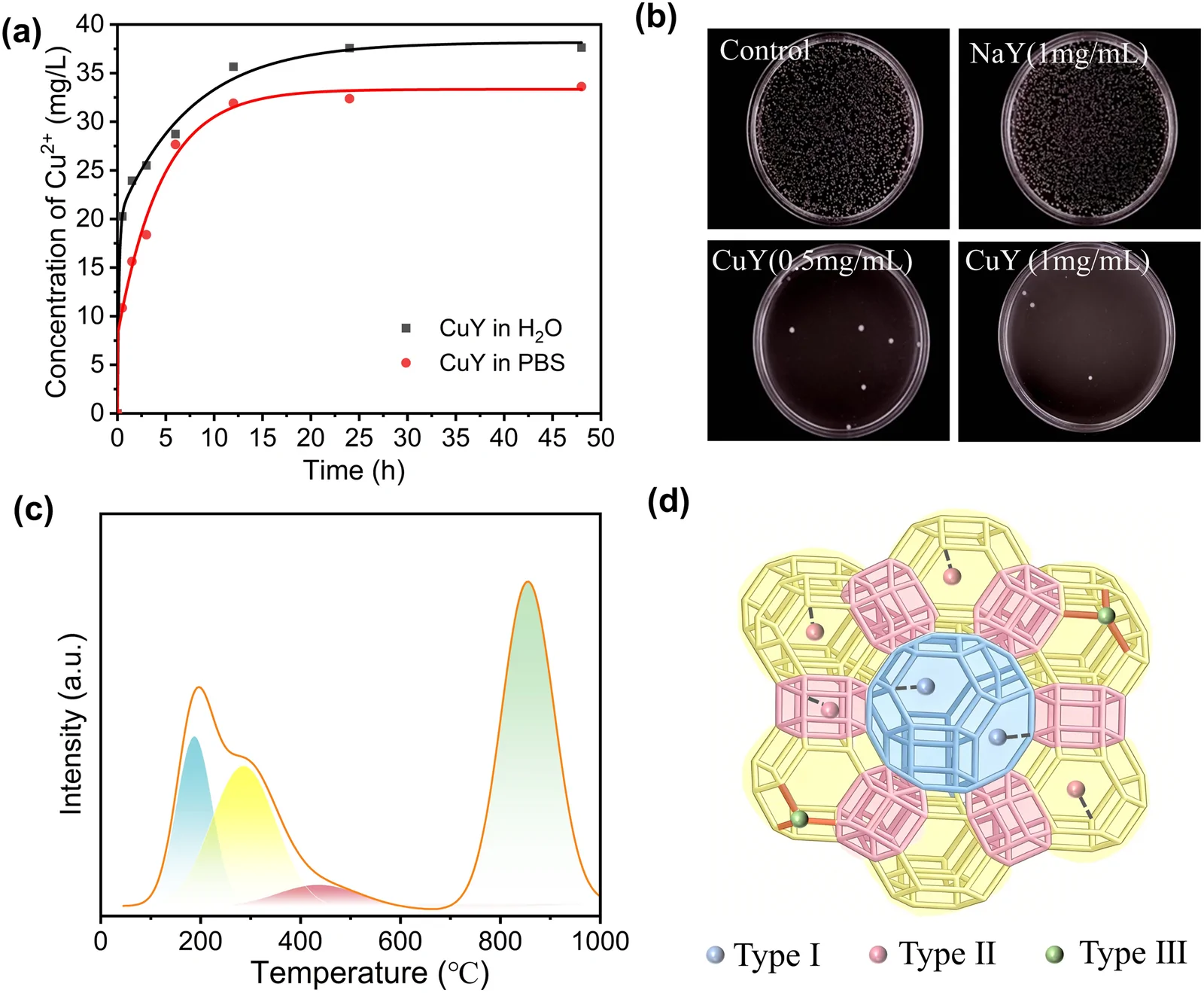
Cu^2+^ release, H_2_-TPR analysis, and antibacterial activity of CuY. (a) Cu^2+^ release profiles in deionized water and PBS under stirring at 37 °C. (b) Photographs of agar plates showing antibacterial activity against *S. aureus* after treatment with NaY and CuY. (c) H_2_-TPR profile with Gaussian deconvolution. (d) Schematic showing three types of Cu^2+^ location in the FAU framework.

To understand how this burst release affects antibacterial performance, we tested CuY against both Gram-positive (*S. aureus*, Fig. 2b) and Gram-negative (*E. coli*, Fig. S3) bacteria using the plate counting method. CuY showed a clear concentration-dependent antibacterial effect. At 1 mg mL^−1^, the number of viable colonies on the agar plates dropped sharply, giving an antibacterial rate above 99.9% for both strains. In contrast, the parent NaY zeolite showed no antibacterial activity at all, confirming that the bactericidal effect comes entirely from the Cu^2+^ ions released from the zeolite.

The strong antibacterial activity of CuY comes from the rapid release of Cu^2+^ in the initial burst. However, this burst release also limits the duration of antibacterial action and raises biocompatibility concerns. To understand the origin of this burst release, we analyzed the distribution of copper species within CuY by H_2_-TPR analysis.

#### 3.1.3 H_2_-TPR analysis and Cu^2+^ release mechanism

As shown in Fig. 2c, the H_2_-TPR profile of CuY can be fitted into four reduction peaks. Based on previous reports, these peaks correspond to copper species in different positions: supercages, sodalite cages/hexagonal prisms, and framework-associated Cu–O–Al linkages.^50–52^ The peak at ∼190 °C is assigned to Cu^2+^ in the supercages (Type I). These ions are weakly bound and desorb easily during both TPR and release. The peaks at ∼290 and 430 °C correspond to Cu^2+^ located in sodalite cages and hexagonal prisms (Type II), where the narrower channels and stronger electrostatic interaction with the AlO_4_^−^ framework make reduction and release more difficult. The peak at ∼850 °C is due to copper species tightly bound to framework oxygen atoms, forming Cu–O–Al linkages (Type III). These species do not desorb under the conditions used in TPR or release experiments.

From the peak areas, the proportions of Type I, Type II, and Type III Cu^2+^ were determined to be 14%, 36%, and 50%, respectively. This matches well with the release data in Fig. 2a, where about 50% of the total copper was released, confirming that the released Cu^2+^ comes almost entirely from Type I and Type II, while Type III remains locked in the zeolite framework and does not contribute to the solution Cu^2+^ concentration. Such quantitative H_2_-TPR analysis of Cu species distribution has been widely applied to characterize copper-exchanged Y zeolites in catalysis.^52^

Taken together, the three-stage release behavior can be explained by the different locations of Cu^2+^ in the FAU framework. As shown in Fig. 2d, Type I Cu^2+^ sits in the large supercages (window size ∼0.74 nm), where they face little resistance to diffusion and only weak electrostatic attraction to the framework. These ions were released quickly in the first 3 h, causing the initial burst. Type II Cu^2+^ is trapped in the much smaller sodalite cages and hexagonal prisms (window size ∼0.23 nm). The tight space and stronger electrostatic binding slow down their release, giving the sustained release phase from 3 to 12 h. Type III Cu^2+^ is covalently bonded to the framework oxygen atoms. These ions are not released under physiological conditions and remain in the zeolite after the plateau is reached.

In short, these results show that the burst release of Cu^2+^ comes mainly from Type I copper in the supercages. While this gives CuY strong short-term antibacterial activity, it also shortens the effective release time and raises safety concerns. To solve this problem, we embedded CuY in a hydrogel matrix to create a second diffusion barrier.

### 3.2 Characterization and *in vitro* performance of CuY@SA composite hydrogel

#### 3.2.1 Morphology and rheological properties

By embedding CuY within a Ca^2+^-crosslinked alginate hydrogel, we built a dual-barrier system, CuY@SA. The internal structure of the freeze-dried CuY@SA hydrogel was examined by SEM. As shown in Fig. 3a, the hydrogel has a porous, interconnected network with irregular pores ranging from several micrometers to tens of micrometers in size. CuY particles were well dispersed and embedded within the pore walls, with no visible agglomeration. The inset of Fig. 3a shows a photograph of the as-prepared CuY@SA hydrogel disc, which is flexible, off-white, about 15 mm in diameter and 2 mm thick.

**Fig. 3:**
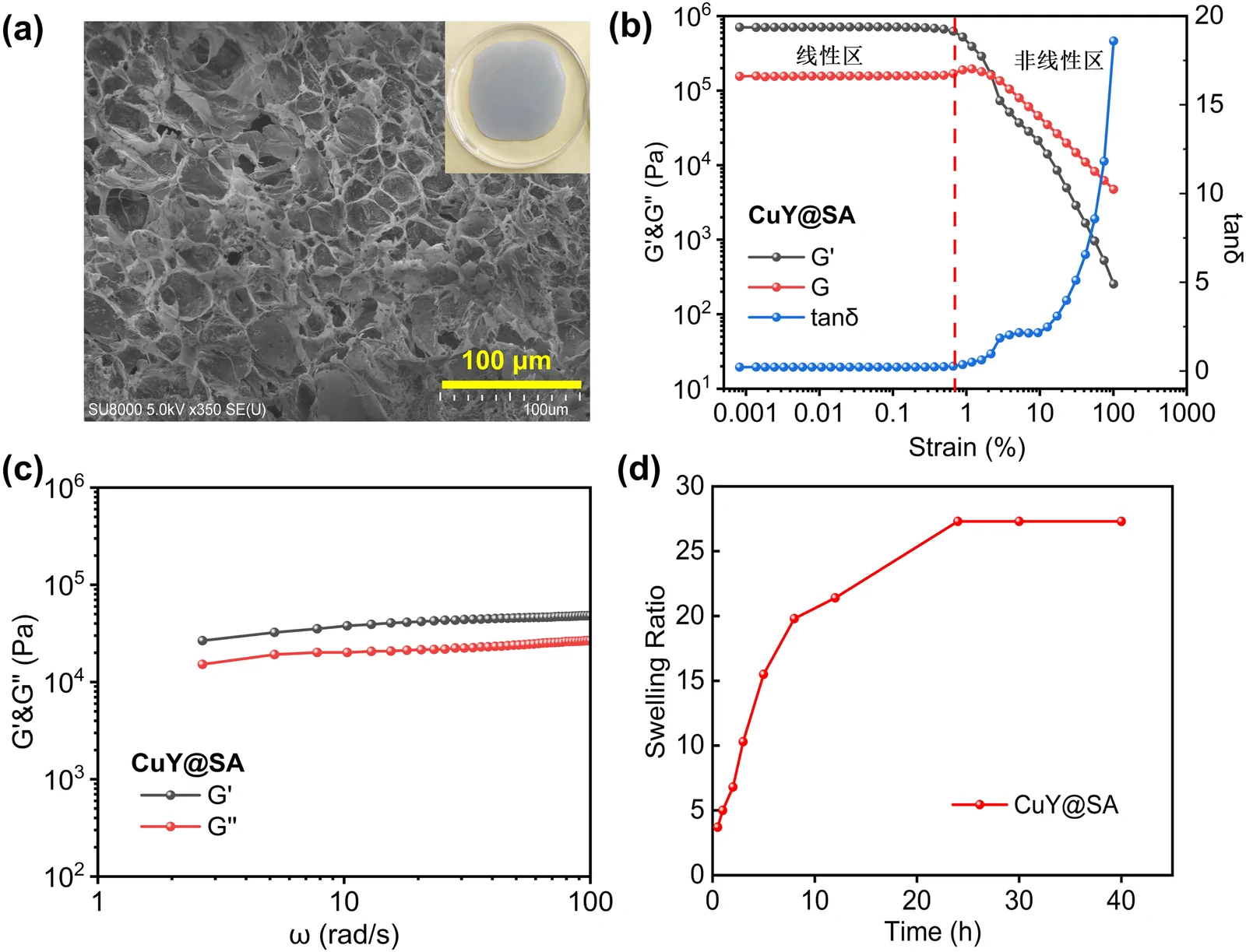
Structure, rheology, and swelling of CuY@SA hydrogel. (a) SEM image of the lyophilized hydrogel showing the porous internal structure. Inset: photograph of the as-prepared CuY@SA hydrogel disc. (b) Strain sweep curves and (c) frequency sweep curves (*G*′ and *G*″) of CuY@SA. (d) Swelling ratio of CuY@SA in PBS (pH 7.4) at 37 °C over time.

The viscoelastic properties of the hydrogels were evaluated by oscillatory rheology. In the strain sweep (Fig. 3b), *G*′ and *G*″ remained constant at low strain (*γ* < 1%), with *G*′ always greater than *G*″, indicating a stable elastic network. As strain increased, *G*′ decreased and eventually crossed *G*″ at around 15% strain, marking the yield point where the gel network begins to break down. The yield stress was about 450 Pa, showing that CuY@SA has good mechanical strength. Compared with the blank SA hydrogel (Fig. S4), the addition of CuY particles broadened the linear viscoelastic region and raised the yield stress. This can be attributed to two factors: CuY particles act as physical fillers that help to bear mechanical stress, and a small amount of Cu^2+^ released during preparation may provide extra ionic crosslinking.^43^ In frequency sweep tests (Fig. 3c) performed at 0.01% strain over 1–100 rad s^−1^, *G*′ remained above *G*″ across the whole range, and both moduli were nearly independent of frequency. This confirms that CuY@SA behaves as a stable elastic gel with no obvious relaxation. These rheological parameters (storage modulus *G*′, loss modulus *G*″ and critical strain) reflect the viscoelastic properties and deformation-resistant capacity of our soft, high-water-content hydrogel, which is critical for its practical performance as a conformable wound dressing. For compact cast hydrogel systems, indentation-based Shore A hardness provides an alternative mechanical metric;^47^ however, this technique is not applicable here because indentation probes would damage the swollen gel network and yield irreproducible measurements.

The swelling behavior of CuY@SA was measured by immersing dried samples in PBS (pH 7.4) at 37 °C. As shown in Fig. 3d, the hydrogel absorbed water rapidly in the first 10 h and reached equilibrium at about 25 h, with a final swelling ratio of approximately 28 g g^−1^. This high water uptake is advantageous for wound dressings, as it helps keep the wound moist and can absorb excess exudate.

#### 3.2.2 *In vitro* antibacterial activity and cytocompatibility

To align with the *in vivo* experimental groups, SA hydrogel (blank control), Cu@SA hydrogel, and CuY@SA hydrogel were evaluated for their *in vitro* antibacterial performance and biocompatibility. The antibacterial activity was tested against *S. aureus* (Fig. 4a) and *E. coli* (Fig. 4b) using the plate counting method. The SA group showed abundant colonies, similar to the negative control, confirming that alginate alone has no antibacterial effect. In contrast, both Cu@SA and CuY@SA strongly inhibited bacterial growth. The antibacterial efficacy of CuY@SA was comparable to that of Cu@SA, indicating that embedding CuY in the hydrogel did not weaken its bactericidal action.

**Fig. 4:**
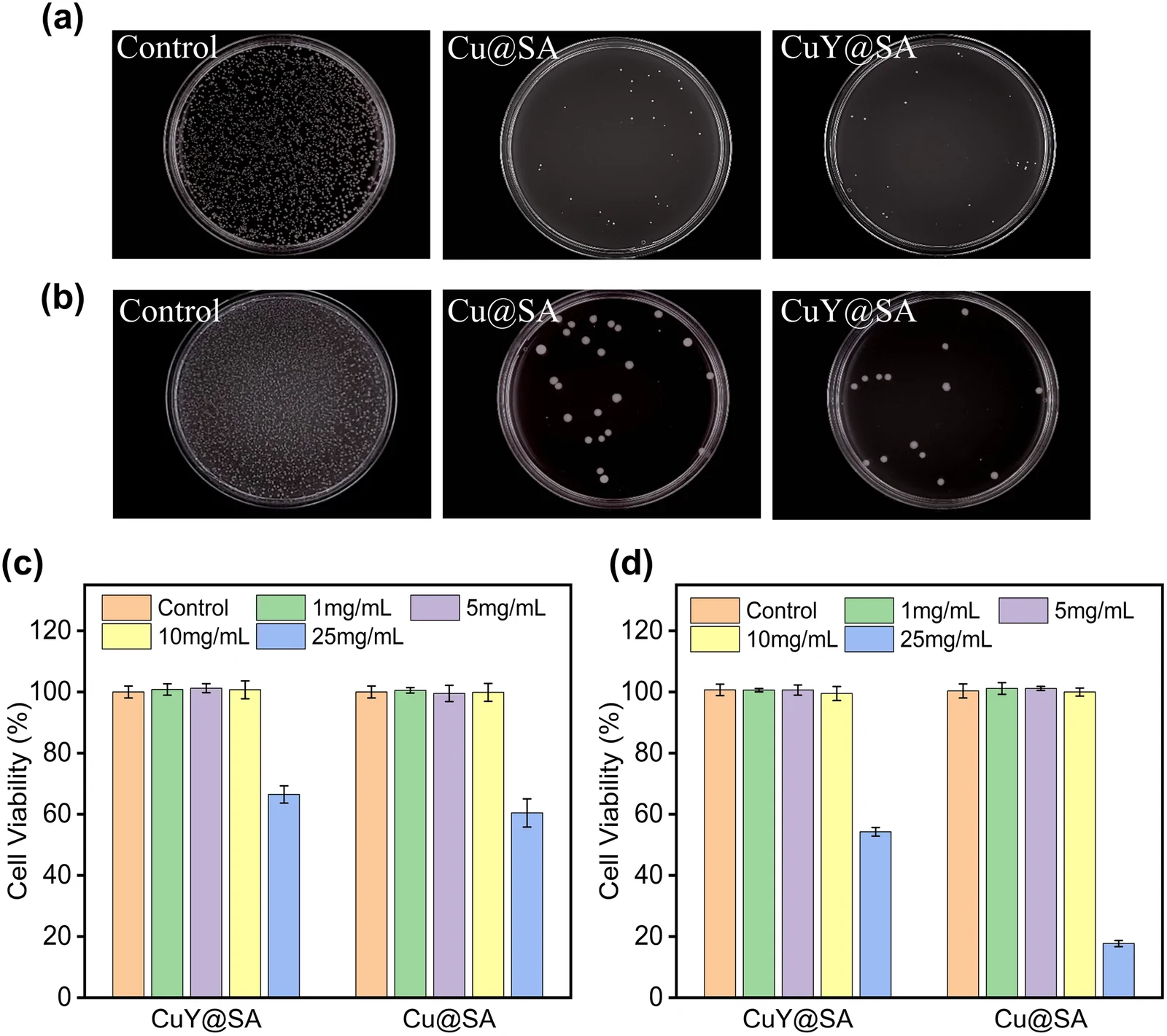
*In vitro* antibacterial activity and cytocompatibility. Photographs of agar plates showing antibacterial activity of SA, Cu@SA, and CuY@SA against (a) *S. aureus* and (b) *E. coli*. Cell viability of NIH-3T3 cells after incubation with hydrogel extracts at different concentrations for (c) 24 h and (d) 48 h.

The cytocompatibility of CuY@SA was evaluated by culturing NIH-3T3 fibroblasts with hydrogel extracts at different concentrations (1, 5, 10, and 25 mg mL^−1^) for 24 h and 48 h, and cell viability was measured by the CCK-8 assay. As shown in Fig. 4c and d, both Cu@SA and CuY@SA showed good cytocompatibility at concentrations up to 10 mg mL^−1^, with cell viability above 98%. At 25 mg mL^−1^, however, the two materials showed markedly different results. Cu@SA caused a sharp drop in viability, with viability dropping to approximately 18% at 48 h. In contrast, CuY@SA retained around 54% viability under the same conditions. This difference demonstrates that pre-loading Cu^2+^ in the zeolite, rather than adding free Cu^2+^ directly into the hydrogel, effectively reduces the toxicity caused by uncontrolled copper release. Overall, CuY@SA combines strong antibacterial activity with favorable cytocompatibility within the relevant concentration range, supporting its further evaluation in animal studies.

#### 3.2.3 Dual sustained release of CuY@SA

To understand how the zeolite carrier affects Cu^2+^ release, we compared the release profiles of CuY@SA and Cu@SA in PBS (pH 7.4) at 37 °C over 48 h. As shown in Fig. 5a, the two hydrogels showed distinctly different release behavior. For Cu@SA, the Cu^2+^ concentration rose quickly and peaked around 6 h, then declined slowly. The total amount of Cu^2+^ released from Cu@SA was much lower than that from CuY@SA. This can be explained by the different state of Cu^2+^ in the two systems. When free Cu^2+^ ions are mixed directly with the alginate solution in the preparation of Cu@SA, most of them become crosslinked with the carboxylate groups, locked inside the network and unable to be released. Only a small fraction, those weakly adsorbed or physically trapped in the pores, can diffuse out in the early stage. In addition, once released, Cu^2+^ can react with phosphate ions in PBS to form precipitates, which further lowers the measured Cu^2+^ concentration after 6 h.

**Fig. 5:**
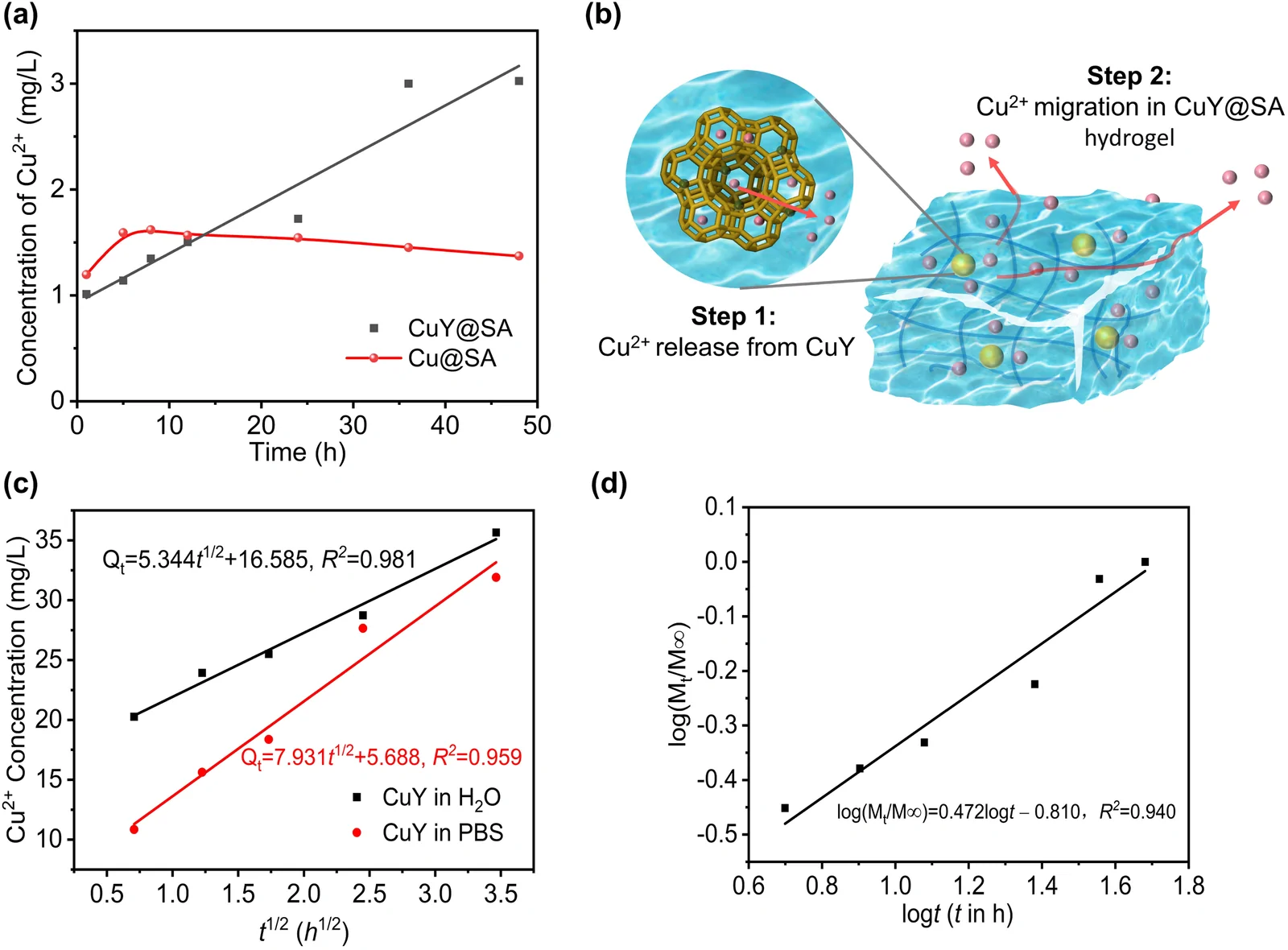
Dual sustained release of CuY@SA. (a) Cu^2+^ release profiles of Cu@SA and CuY@SA in PBS at 37 °C. (b) Schematic of the dual-barrier release mechanism. (c) Higuchi model fitting of Cu^2+^ release from CuY powder in H_2_O and PBS. (d) Korsmeyer–Peppas fitting of Cu^2+^ release from CuY@SA in PBS.

Interestingly, CuY@SA showed a different pattern. Apart from a small release in the first hour, the Cu^2+^ concentration increased nearly linearly from 1 to 48 h. This is different from the burst release of bare CuY powder. The sustained, linear release from CuY@SA reflects the dual-barrier design of this system (Fig. 5b). The first barrier is the zeolite itself. As described before, Cu^2+^ ions sit in three types of sites: supercages (Type I), sodalite cages and hexagonal prisms (Type II), and Cu–O–Al linkages (Type III). Type I and Type II ions must first leave their binding sites and diffuse through the zeolite channels before they can enter the hydrogel. The second barrier is the alginate network, which adds extra resistance and slows the transport of Cu^2+^ into the surrounding solution. Thus, the dual “zeolite–hydrogel” barriers turn the original burst release into a gradual, linear release, extending the effective release time and keeping the local Cu^2+^ concentration within a safe window.

To quantitatively analyze the release mechanism of Cu^2+^ ions, the Higuchi model^53^ and the Korsmeyer–Peppas model^54,55^ were employed to fit the release data (the details are shown in S3 of SI). The release from Cu@SA increased briefly and then declined due to precipitation, so it does not meet the basic assumption of either model and could not be fitted meaningfully. In contrast, the release of Cu^2+^ from CuY powder in both H_2_O and PBS followed the Higuchi model well (Fig. 5c, *R*^2^ = 0.981 and 0.959), indicating Fickian diffusion through the zeolite pores. For CuY@SA in PBS, the release followed the Korsmeyer–Peppas model (Fig. 5d, *R*^2^ = 0.940) with a diffusion exponent *n* = 0.472. This *n* value falls between 0.45 and 0.89, indicating non-Fickian diffusion. This result quantifies the synergistic effect between zeolite and hydrogel. Cu^2+^ dissociates from the zeolite channels (diffusion-controlled) and then moves through the hydrogel network (barrier-controlled). The coupling of these two steps makes the release deviate from pure Fickian behavior.

### 3.3 *In vivo* wound healing evaluation

#### 3.3.1 Wound closure and *in vivo* antibacterial efficacy

The *in vitro* release study showed that CuY@SA suppressed the burst release and extended Cu^2+^ delivery to over 50 h. To test whether this improves healing *in vivo*, we used a mouse model of full-thickness infected skin wounds. Representative wound photographs are shown in Fig. 6a, and the healing rates are plotted in Fig. 6b. Over the 14 days observation period, wounds in all three groups became smaller over time, but the healing rates differed considerably. The control group healed the slowest. The Cu@SA group showed faster closure. CuY@SA gave the fastest and most complete healing, reaching nearly 70% by day 6 and over 98% by day 14, when the wounds were almost fully closed. In contrast, a clear unhealed area remained in the control group. To evaluate *in vivo* antibacterial activity, wound exudate was collected with sterile swabs on day 7, diluted, and plated for colony counting. As shown in Fig. 6c, the CuY@SA group had substantially fewer bacterial colonies than the control and Cu@SA groups. These results confirm that CuY@SA not only speeds up wound closure but also effectively controls local bacterial infection.

**Fig. 6:**
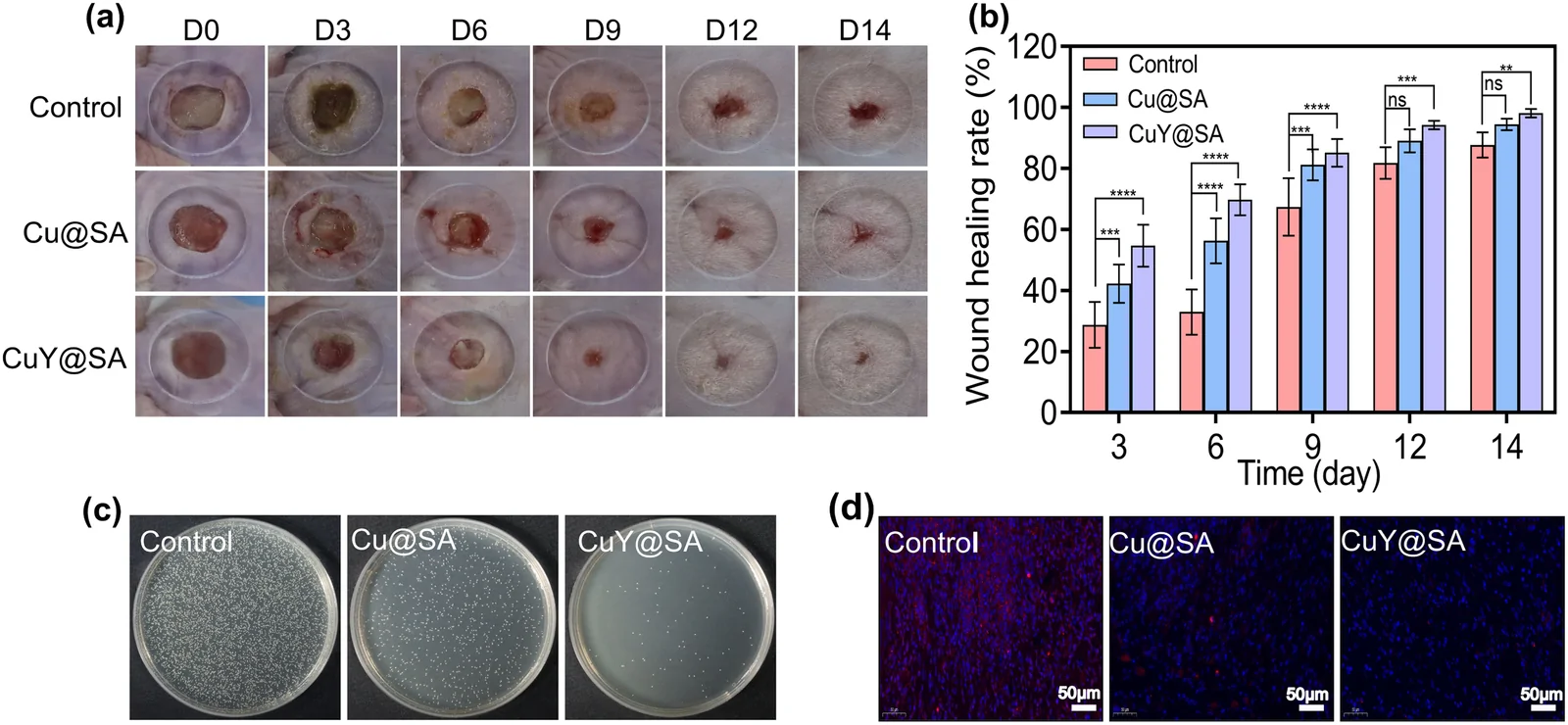
*In vivo* wound healing, antibacterial efficacy, and cuproptosis evaluation. (a) Representative photographs of infected wounds in the control, Cu@SA and CuY@SA groups on days 0, 3, 6, 9, and 14. (b) Wound healing rates over time. (c) Photographs of bacterial colonies recovered from wound swabs on day 7. Plates shown are at 10^5^-fold dilution. (d) Immunofluorescence staining of FDX1 (red) and DAPI (blue) in wound tissue sections on day 14. Data are mean ± SD (*n* = 3). *P < 0.05, **P < 0.01, ***P < 0.001, ****P < 0.0001.

The superior *in vivo* performance of CuY@SA can be attributed to its dual sustained-release design. In Cu@SA, most Cu^2+^ ions are locked inside the alginate network by ionic crosslinking and are unavailable for release, so antibacterial action is weak and short-lived. In CuY@SA, Cu^2+^ is released steadily from the zeolite over an extended period, continuously supplying antibacterial ions while keeping the local concentration within a safe range. This design achieves a good balance between antibacterial activity and tissue compatibility.

#### 3.3.2 Histological and inflammatory evaluation

On day 14, wound tissues were collected for histological analysis. Hematoxylin and eosin (H & E) staining was used to assess re-epithelialization, inflammatory infiltration, and overall tissue regeneration. As shown in Fig. 7a, the control group showed incomplete re-epithelialization with a discontinuous epidermal layer and heavy inflammatory cell infiltration in the dermis, indicating persistent inflammation and delayed healing. The Cu@SA group showed improved epithelial regeneration, with a more continuous epidermal layer and reduced inflammation. The CuY@SA group displayed the best healing outcomes. A complete and continuous epidermal structure was observed, along with regenerated hair follicles and sebaceous glands. Only a few inflammatory cells remained in the dermis, suggesting that tissue remodeling was nearly complete and the skin structure had largely returned to normal.

**Fig. 7:**
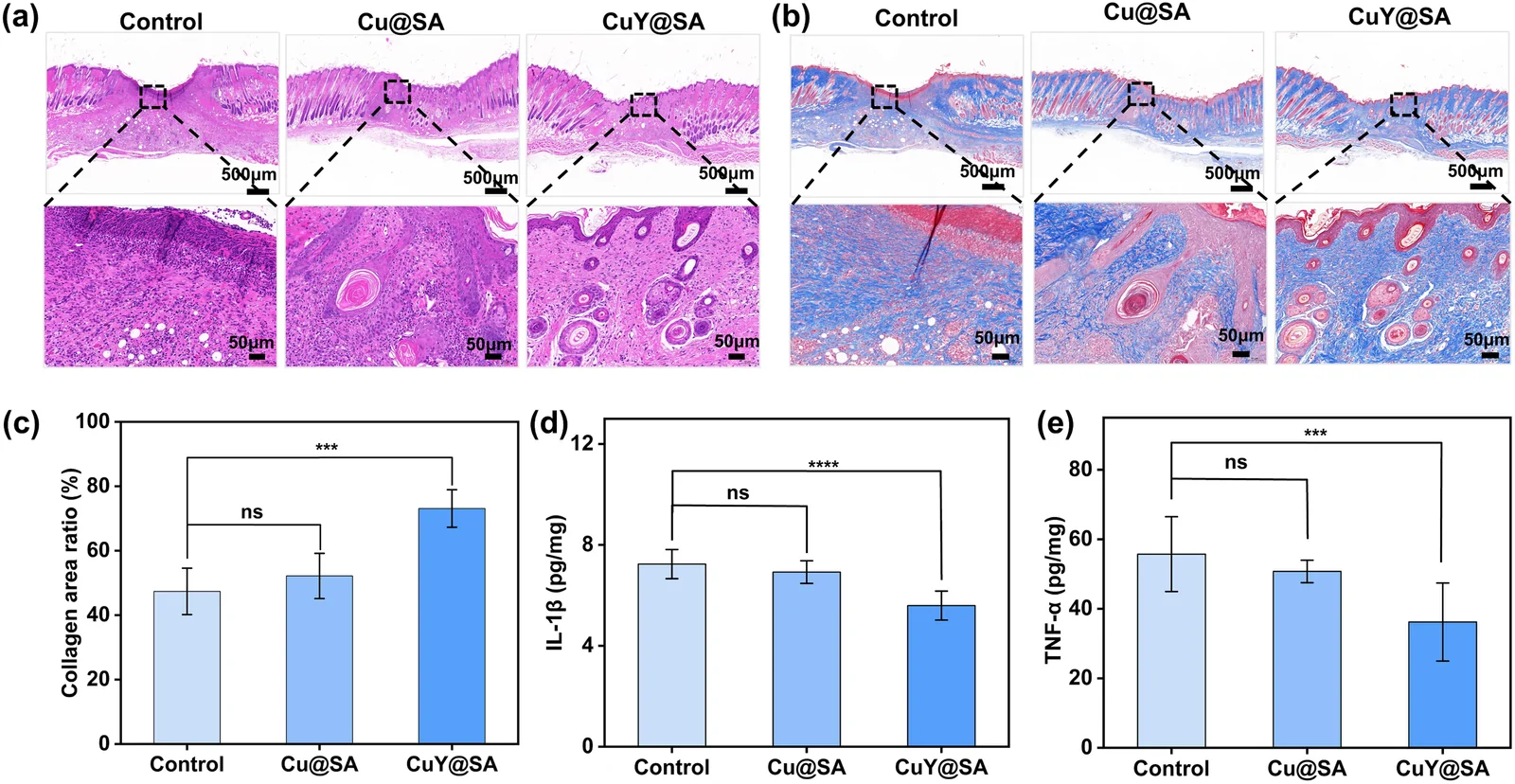
Histological and inflammatory evaluation of wound healing on day 14. (a) H & E staining of wound tissue sections. (b) Masson's trichrome staining showing collagen deposition (blue) and arrangement. (c) Quantitative analysis of collagen area fraction. (d) ELISA quantification of IL-1β and (e) TNF-α levels in wound tissue homogenates. Data are mean ± SD (*n* = 3). *P < 0.05, **P < 0.01, ***P < 0.001, ****P < 0.0001.

Masson's trichrome staining was used to examine collagen deposition and organization. As shown in Fig. 7b, the control group had sparse and disorganized collagen fibers, reflecting poor matrix remodeling. The Cu@SA group showed more collagen with slightly better alignment. The CuY@SA group, however, showed dense and well-organized collagen fibers with a wavy pattern closely resembling native skin. Quantitative analysis (Fig. 7c) confirmed that the collagen area fraction in the CuY@SA group (about 73%) was significantly higher than that in the Cu@SA group (about 52%) and the control group (about 47%). This suggests that the sustained release of Cu^2+^ not only controlled infection but also promoted fibroblast activity and extracellular matrix remodeling.

The levels of pro-inflammatory cytokines IL-1β and TNF-α in wound tissue were measured by ELISA on day 14. As shown in Fig. 7d and e, both Cu@SA and CuY@SA groups had significantly lower cytokine levels than the control group, and the CuY@SA group showed the greatest reduction, approaching the levels found in normal skin. The lower levels of IL-1β and TNF-α suggest that the antibacterial effect of CuY@SA reduced the bacterial load in the wound, which in turn lessened the inflammatory response. This explains why the wound moved more quickly from the inflammatory phase to proliferation and remodeling.

#### 3.3.3 Biosafety evaluation

Three safety aspects were evaluated: local copper accumulation, activation of the cuproptosis pathway, and major organ toxicity. On day 14, the copper content in wound skin tissue was measured by ICP-OES. The CuY@SA group showed a slightly higher copper level than the control and Cu@SA groups, but the value remained within a physiologically acceptable range and well below the known toxic threshold (Fig. S5). To check whether the released Cu^2+^ triggered cuproptosis, a copper-dependent cell death pathway, we stained wound tissue sections for ferredoxin 1 (FDX1), a key regulator of this process.^56^ As shown in Fig. 6d, the control group showed clear FDX1-positive signals, likely due to cellular stress from the persistent bacterial infection. In contrast, the CuY@SA group showed mostly negative FDX1 staining, similar to normal skin, indicating that the Cu^2+^ released from CuY@SA did not activate the cuproptosis pathway and that the local Cu^2+^ concentration stayed within a safe range. H & E staining of major organs (heart, lung, kidney) showed no histopathological abnormalities in any group (Fig. S6). These results, combined with the cytocompatibility data, demonstrate that CuY@SA exhibits excellent *in vivo* biocompatibility and does not cause local or systemic toxicity.

## 4. Conclusions

In this work, a dual-barrier sustained-release antibacterial hydrogel (CuY@SA) was constructed by incorporating Cu-exchanged Y zeolite (CuY) into calcium-crosslinked sodium alginate. The zeolite serves as the first release-regulating unit *via* intrinsic ion-exchange sites, while the alginate network provides a secondary physical diffusion barrier. Combined, they effectively suppress the initial Cu^2+^ burst release and achieve near-linear copper-ion delivery over 48 h. *In vitro* experiments demonstrated that CuY@SA achieves >99.9% bactericidal activity against *E. coli* and *S. aureus*, while retaining favorable cytocompatibility towards NIH-3T3 fibroblasts. In a mouse full-thickness infected-wound model, CuY@SA promoted over 98% wound closure within 14 days, enhanced collagen deposition and re-epithelialization, mitigated local inflammation, and exhibited negligible organ toxicity or cuproptosis activation. Compared with Cu@SA hydrogel with directly incorporated free Cu^2+^, CuY@SA displayed lower cytotoxicity and superior *in vivo* antibacterial performance, verifying the essential role of zeolite-mediated controlled-release behavior. This study presents a feasible strategy that integrates inorganic zeolite carriers with biopolymer hydrogels for infected-wound management. Owing to the tunable ion-exchange features of zeolites, this dual-barrier concept could be extended to other bioactive metal ions and hydrogel platforms for broader biomedical applications.

## Conflicts of interest

There are no conflicts to declare.

## Supplementary Material

RA-OLF-D6RA08040E-s001

## Data Availability

The data supporting this article have been included as part of the supplementary information (SI). Additional data are available from the corresponding author upon reasonable request. Supplementary information is available. See DOI: https://doi.org/10.1039/d6ra08040e.
