# Supplementary material for: A dual-barrier sustained-release hydrogel based on Cu-exchanged Y zeolite and sodium alginate for infected wound healing
Source: RSC Adv. 2026 Nov 12. Online ahead of print. doi: 10.1039/d6ra08040e (PMC13757422; doi:10.1039/d6ra08040e)
Supplement: Supplementary file 1 [file RA-OLF-D6RA08040E-s001.pdf]

Supplementary Information (SI) for RSC Advances.
This journal is © The Royal Society of Chemistry

Supporting Information for

A Dual-Barrier Sustained-Release Hydrogel Based on Cu-Exchanged Y Zeolite and Sodium Alginate for Infected Wound Healing

Ying Rao [b,#], Xiaofang Yu [c,#], Kunlan Xu [d], Lifeng Zheng [e], Shuhua Pu [b], Na Wen [f], Pengzhao Wang [b,*], Wei Lin [a,g,h,i,*]

a Department of Otolaryngology Head and Neck Surgery, the First Affiliated Hospital of Fujian Medical University, Fuzhou 350005, P. R. China

b. College of Chemical Engineering, Fuzhou University, Fuzhou 350108, P. R. China

c. Department of Anesthesiology, Fuzhou University Affiliated Provincial Hospital, Fuzhou 350116, P. R. China

d. Department of Pathology, Fuzhou University Affiliated Provincial Hospital, Fuzhou 350116, P. R. China

e. Department of Orthopedics, the First Affiliated Hospital of Fujian Medical University, Fuzhou 350005, P. R. China

f. College of Materials Science and Engineering, Fuzhou University, Fuzhou, 350116, P. R. China

g. Department of Otolaryngology Head and Neck Surgery, National Regional Medical Center, Binhai Campus of the First Affiliated Hospital of Fujian Medical University, Fuzhou 350212, China

h. Fujian Institute of Otorhinolaryngology, the First Affiliated Hospital of Fujian Medical University, Fuzhou 350005, China

i. Fujian Clinical Research Center for Difficult Otorhinolaryngologic Diseases, the First Affiliated Hospital of Fujian Medical University, Fuzhou 350005, China

# These two authors contributed equally.

* Corresponding authors: wpz@fzu.edu.cn, guaguatong@126.com

S1. Characterization of CuY

Table S1 Elemental composition of NaY and CuY determined by XRF

Sample	NaY	CuY
SiO_2, wt%	64.31	61.51
Al_2O_3, wt%	22.32	21.72
Na_2O, wt%	13.37	3.61
CuO, wt%	-	13.15
Si/Al, mol/mol	2.43	2.40

Calculation of theoretical CuO content. The AlO_4^- tetrahedra in the Y zeolite framework carry negative charges balanced by cations. Based on charge balance, one Cu^{2+} replaces two Na^+ during ion exchange.

Mole of substituted Na^+:

$$\Delta n_{\mathrm{Na}} = 2 \times \frac{13.37\% - 3.61\%}{61.98\ \mathrm{mol/g}} = 3.15 \times 10^{-3}\ \mathrm{mol/g}$$

Mole of loaded Cu^{2+}:

$$\Delta n_{\mathrm{Cu}} = \frac{1}{2} \times \Delta n_{\mathrm{Na}} = 1.58 \times 10^{-3}\ \mathrm{mol/g}$$

Theoretical CuO content:

$$1.58 \times 10^{-3}\ \mathrm{mol/g} \times 79.55\ \mathrm{g/mol} \times 100\% = 12.57\%$$

The measured CuO content (13.15 wt%) agrees well with the theoretical value (12.57 wt%), confirming that most Cu^{2+} ions sit near the AlO_4^- sites through electrostatic interactions.

Table S2. Texture parameters of NaY and CuY

Sample	NaY	CuY
S_{BET}, m^2/g	581	558
S_{micro}, m^2/g	547	515
S_{meso}, m^2/g	34	43
V_{total}, cm^3/g	0.30	0.31
V_{micro}, cm^3/g	0.26	0.27
V_{meso}, cm^3/g	0.04	0.04

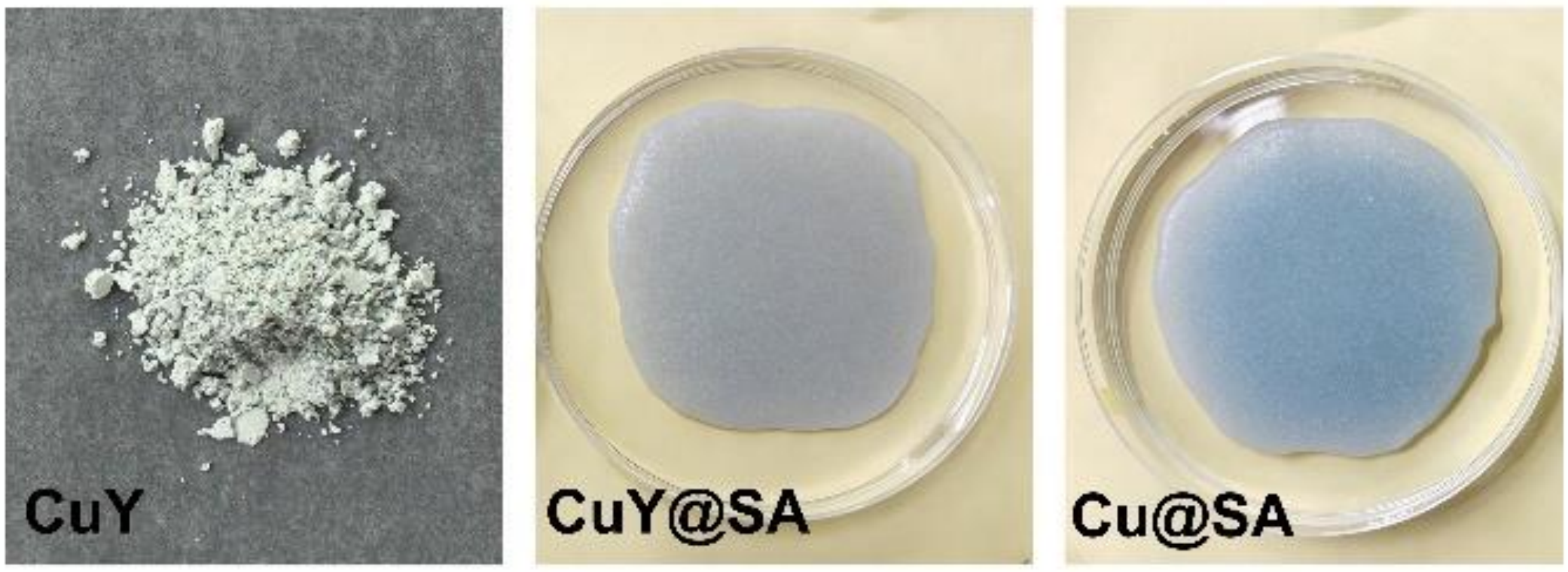

Fig. S1. Images of the as-prepared CuY powder, CuY@SA hydrogel, and Cu@SA hydrogel

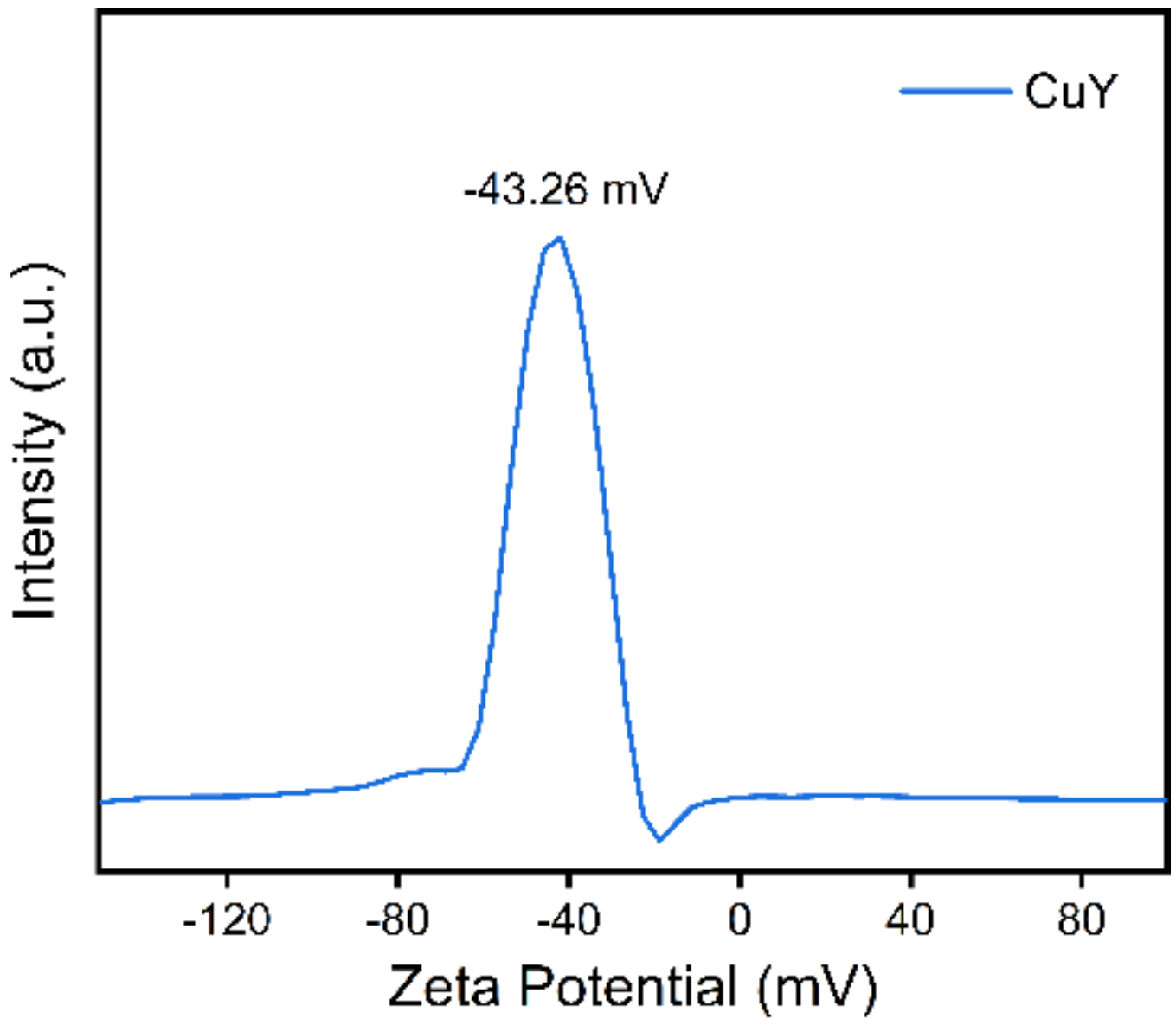

Fig. S2. Zeta potential distribution of CuY particles

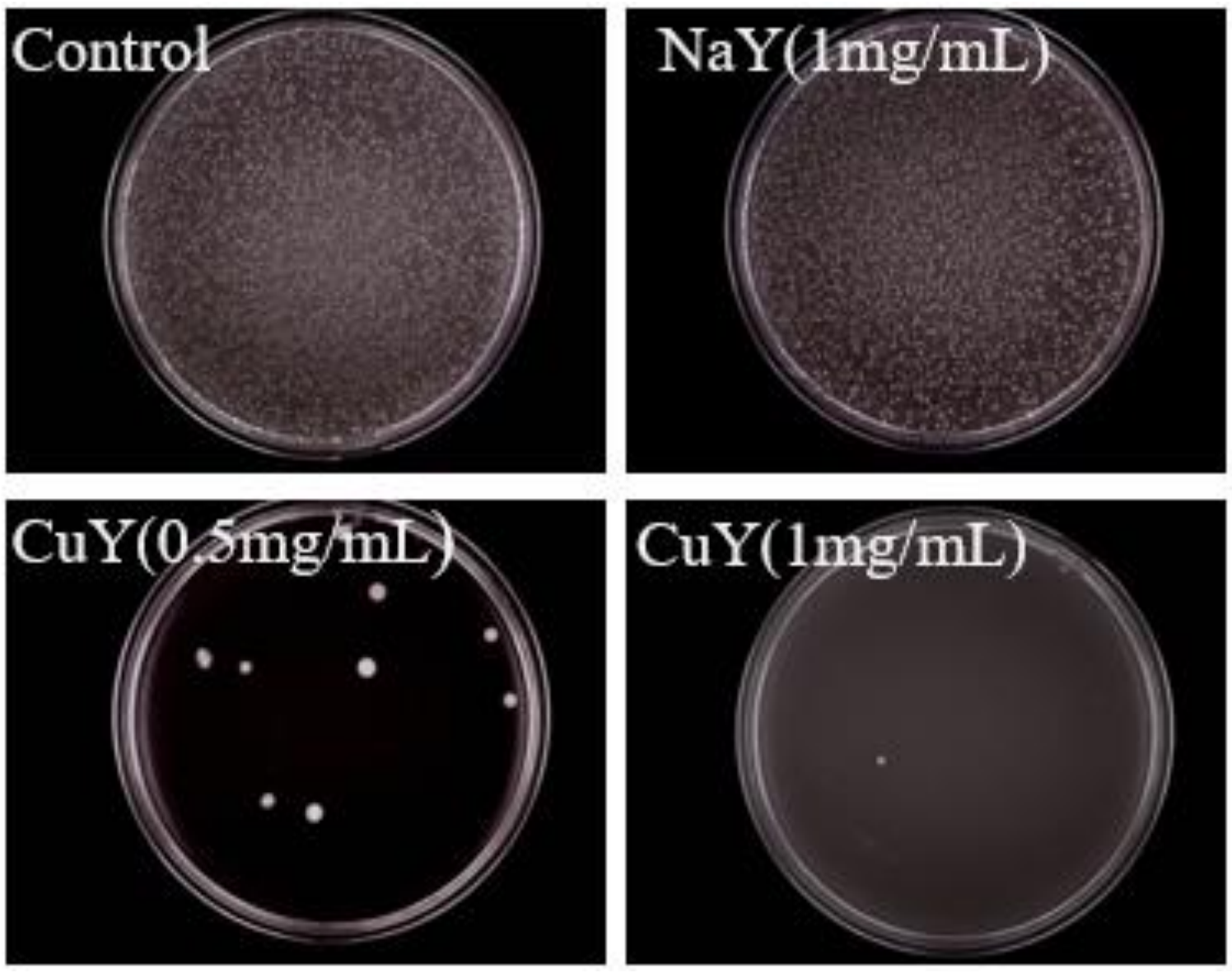

Fig. S3. Photographs of agar plates showing antibacterial activity of NaY and CuY against *E. coli*.

S2. Rheological characterization of SA hydrogel

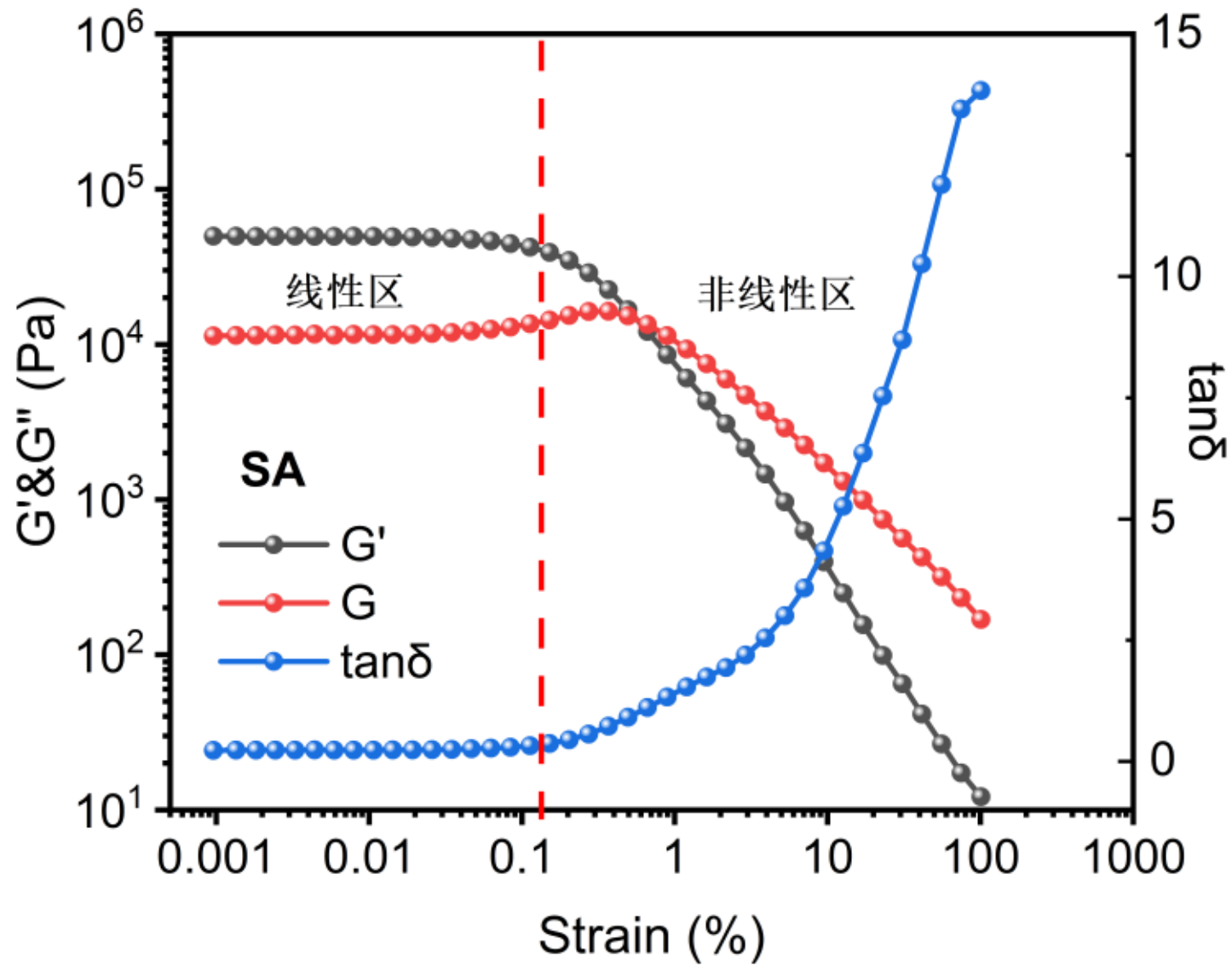

Fig. S4. Strain sweep curves (G′ and G″) of the blank SA hydrogel.

S3. Kinetic modeling of Cu^{2+} release

To further understand the release mechanism, the release data were analyzed using the Higuchi and Korsmeyer-Peppas models.

CuY powder, as a porous inorganic matrix in which Cu^{2+} is dispersed, satisfies the basic assumption of the Higuchi model:

$$Q_t = K_{\mathrm{H}} \cdot t^{1/2} \tag{S1}$$

Where Q_t is the cumulative release amount (mg/L) at time *t*, K_{H} is the Higuchi release rate constant. The release of Cu^{2+} from CuY in both H_2O and PBS followed this model well (Figure 5c, R^2=0.981 and 0.959), confirming Fickian diffusion through the zeolite pores.

For the CuY@SA hydrogel, the Korsmeyer–Peppas model was applied:

$$\frac{M_t}{M_\infty} = K_{\mathrm{KP}} \cdot t^n \tag{S1}$$

Where $\frac{M_t}{M_\infty}$ is the fractional release at time *t*, K_{KP} is the rate constant, and *n* is the diffusion exponent. The fitting (Figure 5d) gave n=0.472 (R^2=0.940). This n value falls between 0.45 and 0.89, indicating a non-Fickian release mechanism. This result quantitatively confirms that Cu^{2+} release is governed by both dissociation from the zeolite channels (diffusion-controlled) and transport through the hydrogel network (barrier-controlled). The coupling of these two processes causes the release to deviate from pure Fickian diffusion. The release data from Cu@SA could not be fitted by either model due to the non-monotonic trend caused by Cu^{2+}–phosphate precipitation.

S4. Supplementary figures for biosafety evaluation

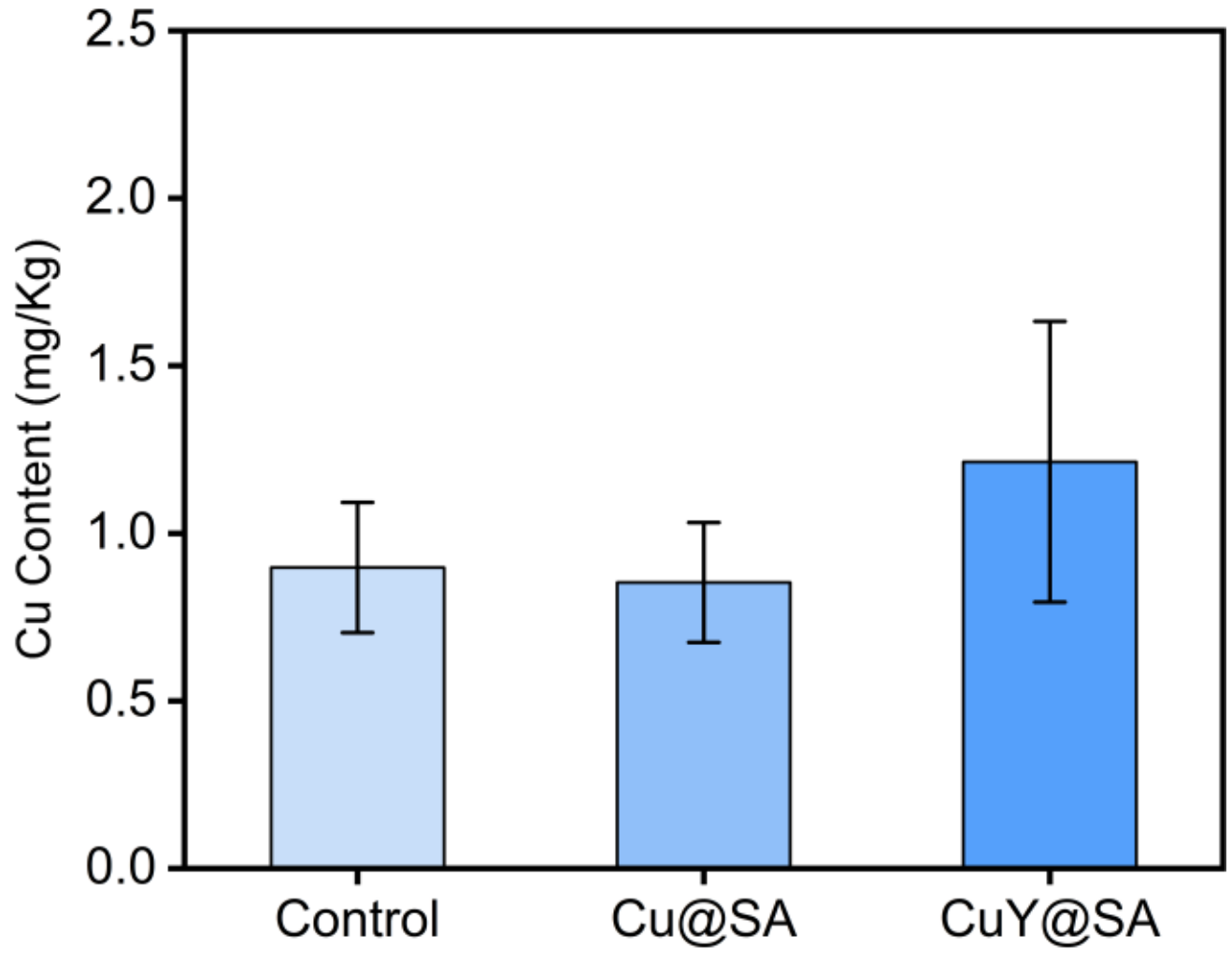

Fig. S5. Copper content in wound skin tissue on day 14 measured by ICP-OES. The CuY@SA group showed a slightly higher copper level than the control and Cu@SA groups, but all values remained within a physiologically acceptable range.

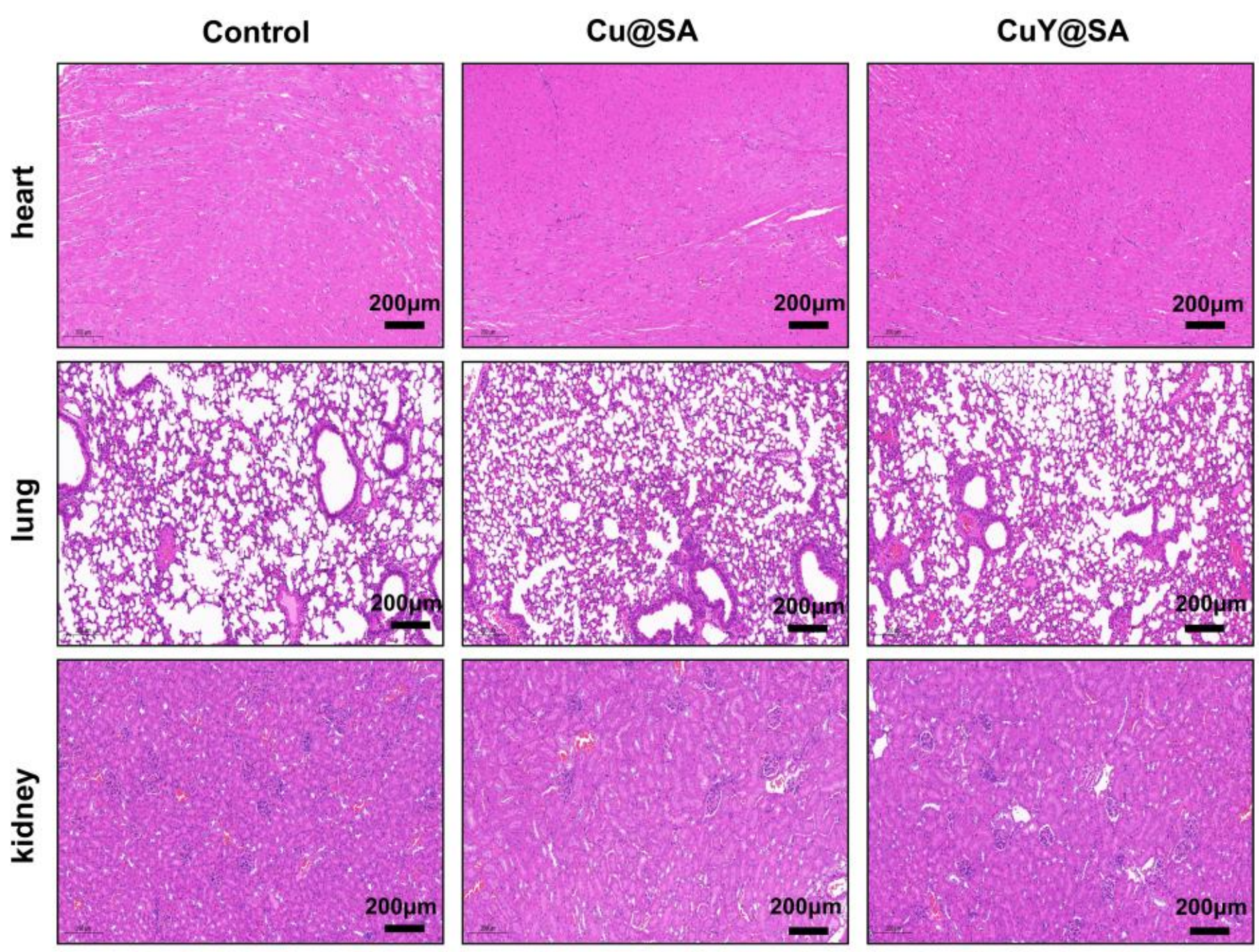

Fig. S6. H&E staining of major organs (heart, lung, kidney) from mice in the control, Cu@SA, and CuY@SA groups on day 14. No histopathological abnormalities were observed in any group.
